# Monoarticular juvenile idiopathic arthritis as a distinct clinical entity A proof-of-concept study

**DOI:** 10.1186/s12969-023-00869-w

**Published:** 2023-08-10

**Authors:** Francesco Zulian, Beatrice Pierobon, Maria Elisabetta Zannin, Caterina Politi, Fabio Vittadello, Alessandra Meneghel, Francesca Tirelli, Giorgia Martini

**Affiliations:** 1 Department of Woman and Child Health, University of Padova, Via Giustiniani 3, Padova, 35128 Italy; 2 Legal Medicine, Caterina Politi, University of Padova, Padua, MD Italy; 3 Explora – Research and Statistical Analysis, Vigodarzere, Italy

**Keywords:** Juvenile idiopathic arthritis, Joint hypermobility, Outcome, Uveitis

## Abstract

**Background:**

Currently, monoarticular Juvenile Idiopathic Arthritis (monoJIA) is included in the ILAR classification as oligoarticular subtype although various aspects, from clinical practice, suggest it as a separate entity.

**Objectives:**

To describe the clinical characteristics of persistent monoJIA.

**Methods:**

Patients with oligoJIA and with at least two years follow-up entered the study. Those with monoarticular onset and persistent monoarticular course were compared with those with oligoJIA. Variables considered were: sex, age at onset, presence of benign joint hypermobility (BJH), ANA, uveitis, therapy and outcome. Patients who had not undergone clinical follow-up for more than 12 months were contacted by structured telephone interview.

**Results:**

Of 347 patients with oligoJIA, 196 with monoarticular onset entered the study and 118 (60.2%), identified as persistent monoJIA, were compared with 229 oligoJIA. The mean follow-up was 11.4 years. The switch from monoarticular onset to oligoarticular course of 78 patients (38.8%) occurred by the first three years from onset. In comparison with oligoJIA, the most significant features of monoJIA were later age at onset (6.1 vs. 4.7 years), lower female prevalence (70.3 vs. 83.4%), higher frequency of BJH (61.9 vs. 46.3%), lower frequency of uveitis (14.4 vs. 34.1%) and ANA+ (68.6 vs. 89.5%) and better long-term outcome.

**Conclusions:**

MonoJIA, defined as persistent arthritis of unknown origin of a single joint for at least three years, seems to be a separate clinical entity from oligoJIA. This evidence may be taken into consideration for its possible inclusion into the new classification criteria for JIA and open new therapeutic perspectives.

**Supplementary Information:**

The online version contains supplementary material available at 10.1186/s12969-023-00869-w.

## Background

Juvenile idiopathic arthritis (JIA) is the most common chronic rheumatic disease of childhood with autoimmune pathogenesis [1]. The ILAR classification, currently in use, includes all arthritis of unknown aetiology that occur before the age of 16 for at least 6 consecutive weeks [2]. It defines seven subtypes of JIA and the oligoarticular one (oligoJIA), defined by the involvement of maximum four joints in the first six months since JIA diagnosis, is the most frequent subtype. Oligo-JIA mainly affects the large joints of the lower limbs, less frequently the joints of the upper limbs [3, 4]. Data from the literature show that, in the majority of patients with oligoJIA, the onset is monoarticular [5] and in many cases the disease evolves into an oligoarticular form [4].

Currently, persistent monoarticular Juvenile Idiopathic Arthritis (monoJIA) is included in the oligoarticular subtype although various aspects, from clinical practice, may suggest considering it as a separate entity.

Our *proof-of-concept* study is aimed to describe the clinical characteristics of persistent monoJIA and to propose clinical criteria that may distinguish it from the oligoarticular form.

## Methods

Patients with a diagnosis of persistent or extended oligoJIA, defined according to the ILAR criteria [2] and with at least two years of follow-up from the disease onset, were included in this retrospective observational study. Patients were distinguished in three groups: patients with monoarticular onset and persistent involvement of a single joint (‘monoJIA’), patients with monoarticular onset but oligoarticular course (‘switch’) and patients with oligoarticular involvement since the onset. Each patient was evaluated using a standardized diagnostic approach and treat-to-target strategy [6] at the onset and subsequently every 3–4 months, depending on the clinical status. Patients in clinical remission or who had not been evaluated for at least 12 months were contacted by telephone, through a structured interview, to investigate the current status of the disease.

For each patient, the following parameters were evaluated: sex, age at onset, site of arthritis at onset and during follow-up, presence of benign joint hypermobility (BJH), antinuclear antibodies (ANA), presence of uveitis, therapy and outcome at the last evaluation. The presence of BJH was defined according to the Beighton criteria, considering a cut-off value for the Beighton score of ≥ 5/9 [7, 8]. ANA were tested at diagnosis and then rechecked every 6–12 months and were considered positive with titer ≥ 1/160 on Hep2 Cells. The ophthalmological screening, to evaluate the presence of anterior uveitis, was performed at the time of diagnosis and then every three months by slit lamp examination. Patients with rheumatoid factor or HLA-B27 positive were excluded.

Therapy was categorized into four levels as follows: intraarticular corticosteroid (IACS) injection; non-steroidal anti-inflammatory drugs and/or oral corticosteroids (AIDs, Anti-Inflammatory Drugs) conventional synthetic disease-modifying anti-rheumatic drugs (csDMARDs) and biological disease-modifying anti-rheumatic drugs (bDMARDs).

Outcome was defined according to Wallace’s criteria [9] as complete clinical remission (CR) or inactive joint and/or ocular disease, in the absence of therapy, for at least twelve continuous months; clinical remission on medication (CRM) or absence of disease activity for at least six continuous months but with therapeutic treatment still in progress; active disease (AD) as the presence of uveitis or definite active arthritis defined as swelling, warmth, and functional limitation in at least one joint.

### Statistical analysis

For each variable considered in the study, the absolute and percentage distributions of the subjects were calculated. For quantitative variables, the main indicators of centrality and variability were calculated.

The diagnostic “persistence” in the monoJIA category was measured with Kaplan Meier curves in all subjects with initial monoarticular onset, considering the time from the initial diagnosis to a possible switch or to the end of follow up. In the Kaplan-Mayer survival analysis, the event “extension of arthritis to a second or more joints” (“switch”) was considered as the outcome while “survival” was considered the persistent involvement of a single joint.

To identify which variables could contribute to the switch from mono to oligoJIA, the data were analyzed according to a binary logistic regression model (backwards method with Wald statistics). This model allows to analyze the asymmetric relationship (dependence) existing between a response variable (“switch”) and one or more explanatory variables (predictors), properly selected (sex, age at onset class, presence of BJH, ANA or uveitis at onset).

**Fig. 1:**
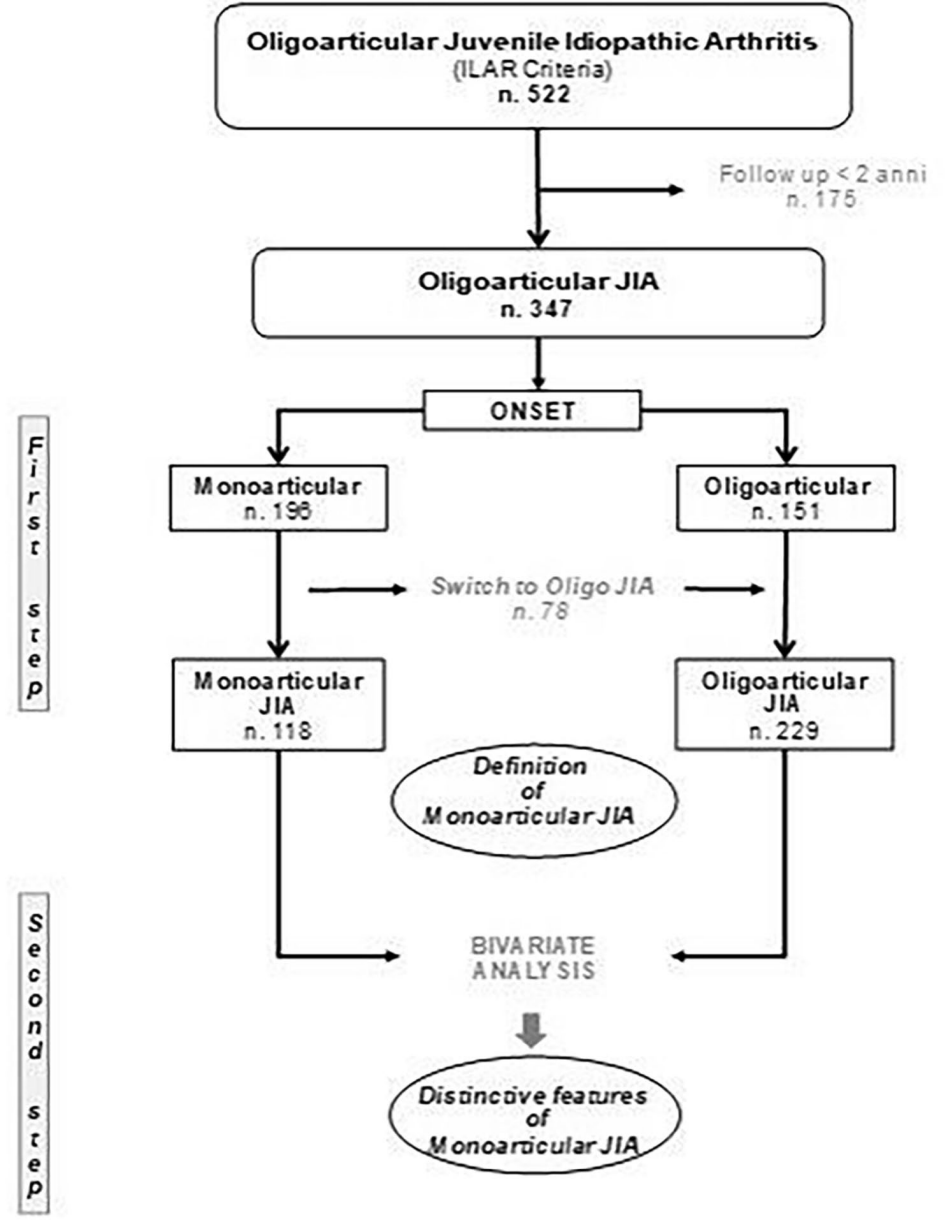
Flow chart of the study design

Patients with persistent monoarticular course (monoJIA) were then compared to those with oligoarticular course (oligoJIA), according to the scheme summarized in Fig. 1, to evaluate the distinctive characteristics of the two groups. Comparison between clinical variables in the two groups was analyzed using non-parametric tests after verifying the normality of the distributions of the variables considered. Mann-Whitney U test and Kruskal-Wallis test were used for numerical variables, and Pearson’s *X*^2^ test and Fisher’s exact test for categorical variables, where appropriate. A p-value less than 0.05 (two-tailed test) was considered statistically significant. All analyzes were performed using IBM SPSS statistical software (Vers. 20.0).

According to the Padua University Hospital policy, approval from the Ethics Committee was not needed because all data were anonymously collected. The study was performed in accordance with the Declaration of Helsinki. Patients consented to participation in the study and publication of data.

## Results

**Fig. 2:**
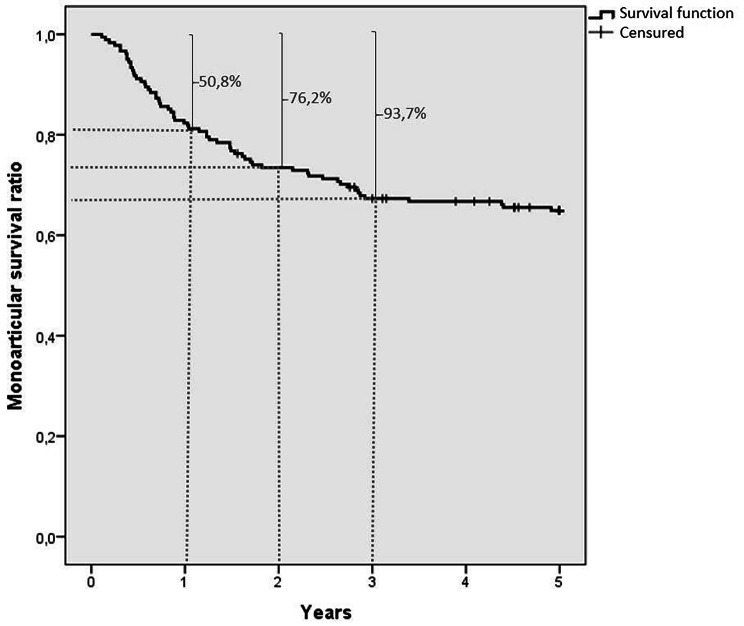
Kaplan-Meier survival curve showing the risk of switch from mono to oligo JIA. Each step of the curve corresponds to the occurrence of one event. The dashes across the curve illustrate censored patients (end of follow up)

Among 522 patients with oligoarticular JIA patients seen at our Center between January 2018 and December 2021, 347 patients had a follow-up longer than two years: 196 (56.5%) had a monoarticular onset during the first 6 weeks of disease, 78 (39.8%) of them subsequently became oligoarticular (“switch”) and 118 maintained a monoarticular course (monoJIA) (Fig. 1). During a mean observation period of 11.4 ± 4.9 years (range 2-25.4 years), we made a retrospective analysis of 17,546 electronic records of rheumatology and ophthalmology visits with an average of 51 visits/patient. In children with monoarticular onset, the 78 switches to oligoJIA occurred almost exclusively within the first three years of disease onset (Fig. 2). In particular, 50.8% within the 1st year, 76.2% within the 2nd year and 93.7% by the 3rd year since the disease onset. Therefore, these data suggest that monoJIA can be defined as such not earlier than three years since the disease onset.

### Risk factors for switch from mono to oligoJIA

**Fig. 3:**
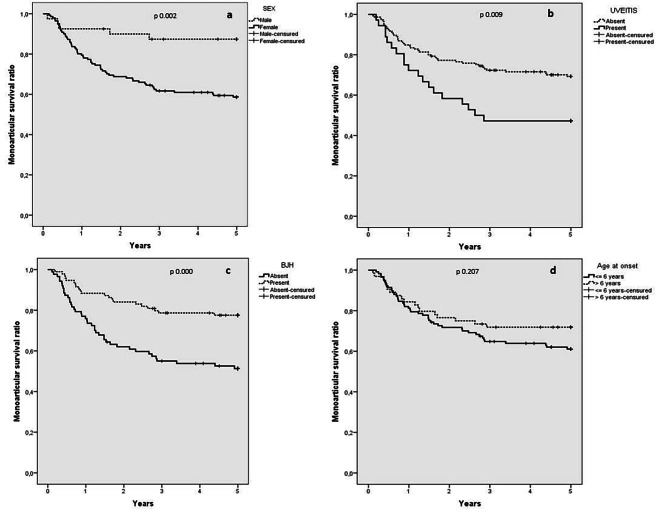
Kaplan-Meier survival curves of the time of switch from Mono to Oligo JIA according to sex (a), presence of uveitis (b), benign joint hypermobility (BJH) (c) and age class (≤ or > 6 years) at onset (d)

In the binary logistic regression analysis, the variables that were found to be significant in differentiating patients with persistent monoJIA from those subject to switch were sex, presence of uveitis and BJH (Fig. 3). Comparison of patients’ survival curves revealed that disease extension to two or more joints was more frequent in females than in males (41.1% vs. 12.5%, p = 0.002) (Fig. 3a). The ocular involvement significantly characterizes the switching as disease extension occurred in 52.8% of patients with uveitis versus 30.3% of those without it (p = 0.009) (Fig. 3b). BJH represents one of the most distinctive characteristics of persistent monoJIAs. In fact, the switch occurred in only 22.3% of BJH + patients versus 48.3% of those BJH- (p = 0.000) (Fig. 3c). Conversely, the age at onset, stratified into two classes, lower or higher than 6 years, was not significant in this contest (p = 0.207) (Fig. 3d), the same for ANA (data not shown).

### Comparison between mono and oligoarticular JIA

The clinical features of patients with persistent monoJIA (n = 118) were then compared to those with oligoJIA (n = 229) (Fig. 1). Interestingly, monoJIA affected almost exclusively the lower limbs (94.1%), particularly the knee (83.9%) and, less frequently, the ankle (10.2%). The upper limbs were affected in six patients (5.1%) and the temporomandibular joint in only one patient (0.8%). As summarized in Table 1, females were more frequently affected in both groups, although with higher rate in oligoJIA than in monoJIA (83.4% and 70.3% respectively, p = 0.004), and with a female-to-male ratio of 2.4:1 in monoJIA and 5.0:1 in oligoJIA.

**Table 1:** Clinical characteristics of Monoarticular and Oligoarticular JIA

			MonoJIA No.118†	OligoJIA No. 229	*p*
**Sex** (F)			83 (70.3)	191 (83.4)	*0.004*
**Age at onset** (years)			6.1 (3.9)	4.7 (3.2)	*0.002*
**Presence of BJH**			73 (61.9)	106 (46.3)	*0.006*
**Uveitis**			17 (14.4)	78 (34.1)	*0.000*
**ANA+**			81 (68.6)	205 (89.5)	*0.000*
**Therapy**		IACS	107 (90.7)	218 (95.2)	*0.102*
		AIDs	60 (50.8)	199 (86.9)	*0.000*
		csDMARD	22 (18.6)	181 (79.9)	*0.000*
		bDMARD	6 (5.1)	83 (36.2)	*0.000*
**Long-term outcome**		CR	97 (82.2)	101 (44.1)	*0.000*
		CRM	15 (12.7)	97 (42.4)	
		AD	6 (5.1)	31 (13.5)	

† Data are number (% or standard deviation)

Legend: AD, active disease; ANA, antinuclear antibodies; CR, clinical remission; CRM, clinical remission on medication; F, female; BJH, benign joint hypermobility; monoJIA, monoarticular juvenile idiopathic arthritis; oligoJIA, oligoarticular juvenile idiopathic arthritis; IACS intra-articular corticosteroids; AIDs anti-inflammatory drugs; csDMARD conventional synthetic disease-modifying anti-rheumatic drugs; bDMARD biological disease-modifying anti-rheumatic drugs

The age at onset of monoJIA was significantly higher than in oligoJIA (mean 6.1 vs. 4.7 years, p 0.002). BJH was associated with monoJIA in 61.9% of cases and in 46.3% of oligoJIA (p = 0.006).

Uveitis was present in only 17 patients with monoJIA (14.4%) and in 9 of them uveitis was already detected at disease onset. In the other eight patients, uveitis was detected after a mean 15.5 months (range 4–26 months) since the disease onset. In oligoJIA, uveitis was reported in a significantly higher rate (34.1% of patients, p 0.000). During the disease course, ANA were detected in most patients of both groups but with a lower frequency in monoJIA (68.6%) than in oligoJIA (89.5%, p = 0.000).

As highlighted in Table 1, a greater use of systemic treatments (AIDs, csDMARDs and bDMARDs) was needed for oligoJIA patients compared to monoJIA (p = 0.000). In contrast, the percentage of subjects undergoing IACS treatment was comparable between the two groups (90.7% vs. 95.2%, p = 0.102). Only 50.8% of patients with monoJIA were treated for brief periods with AIDs, versus 86.9% of oligoJIA. As for second line treatments, only 22 patients (18.6%) with monoJIA needed csDMARDs against 79% of oligoJIA. It is interesting to note that in two-thirds of monoJIA patients on DMARDs, the therapy was aimed to treat uveitis, not arthritis. BDMARDs were used in only six patients with monoJIA (5.1%) and, in all of them, for severe course uveitis.

As for disease outcome, after a mean follow-up of 11.4 years (range 2-25.4 years), we found that, among monoJIA patients, only 5.1% had still active disease at the last evaluation while 12.7% resulted in CRM and as many as 82.2% in CR. Among oligoJIA, on the other hand, only 44.1% of patients were in CR, 42.4% were in CRM and 13.5% in the activity phase (p = 0.000).

## Discussion

Although patients with monoarticular course JIA are currently considered as part of the oligoarticular subtype, they seem to have clinical characteristics somehow different from those with oligoJIA. The present study, with a long-term follow up, seems to confirm this general observation and adds other evidence suggesting that monoJIA may be considered as a separate condition, quite distinct from oligoJIA.

It is well known that oligoJIA has a monoarticular onset in most patients and that lower limbs joints are involved in the majority of them, being the knee the most affected joint (83.9%) followed by ankle (10.2%) and less frequently by other joints (elbow, wrist, and TMJ) [3, 4, 10].

Our study allowed us to evaluate the clinical characteristics of patients in whom from a single joint the disease spread to two or more joints, becoming oligoarticular. This group, defined as “switch”, resulted having the same characteristics as oligoJIA and, compared to monoJIA, had an increased incidence of uveitis, affected more frequently females and was less associated with BJH. Since in our study the switch occurred in 94% of cases within three years of onset, this time interval may represent the time limit for defining a persistent monoJIA.

The comparison between monoJIA with persistent monoarticular course and oligoJIA allowed us to identify the peculiar characteristics of the two groups (Table 1). MonoJIA affects females less frequently than oligoJIA, has a later onset, is less complicated by uveitis but more frequently associated with BJH.

BJH, present in almost two thirds of patients, predominantly affects the lower limbs where the vast majority of monoJIA occurs. These data seem to suggest a possible relevant role of ligament hyperlaxity as a co-causative factor in the pathogenesis of arthritis in monoJIA as compared to oligoJIA, where instead autoimmunity seems to play a major role. In fact, ANA, known markers of autoimmunity, are significantly more frequent in oligo than in monoJIA.

It is well known that BJH is considered a physiological phenomenon in young children [7, 8]. In a previous study, we reported a high prevalence of BJH (63%) among patients with oligoJIA and this frequency was significantly higher than reported by a recent study performed in healthy schoolchildren in the same geographic area (40.5%, p = 0.006) [11, 12]. In the present study, we confirm the high frequency of BJH, particularly in monoJIA. In the literature, subjects with hypermobile joints result having more frequent joint injuries, subluxations, dislocations and other complaints and are more predisposed to arthritis or arthralgia [13–15]. Indeed, other studies have shown that altered contact mechanics and abnormal articular loading in instable joints may cause increased contact stress directional gradients and articular surface incongruity that may lead to articular cartilage disruption and joint inflammation [16]. Therefore, the mechanical component might be a crucial factor in both triggering for the onset of arthritis and in its persistence.

Interestingly, we found a significantly lower incidence of uveitis in monoJIA both at diagnosis and during the disease course (Table 1). This is another important finding as uveitis is a cause of long-term disability and damage in oligoJIA [5, 17, 18]. We do not have a clear-cut explanation for this. However, it might partially confirm the more ‘local mechanical’ nature of monoJIA, with systemic inflammation playing a minor role. Indeed, the presence of uveitis lead to the early use of DMARDs in the majority of monoJIA patients in our cohort. In fact, 6/9 patients with anterior uveitis (AU) already present at the disease onset had severe disease course and are the same ones who were on bDMARDs treatment at the last evaluation. Furthermore, in most monoJIA patients ocular involvement is present already at diagnosis, it unlikely develops later and in any case in a less severe form.

As far as the outcome is concerned, the results confirm the better outcome of monoJIA, in which the majority of patients (82.2%) were in complete clinical remission at the last evaluation. Conversely, only 44.1% of the oligoJIAs were in complete remission, while the majority (55.9%) were either still active or in remission on DMARDs. These data confirm the results of a large long-term study from Norway in which the 7-year remission rate in a population of 201 patients with oligoJIA was 49.2% [19]. Moreover, our data approximate results from two previous studies in oligoJIA patients observed 10 years after the onset, where CR was reported in only 47% and 45% of cases, respectively [20, 21]. According to a study by Wallace et al. who analyzed 258 patients with oligoJIA, disease remission at 4 years follow up was achieved in 68% of cases with persistent oligoJIA and in only 31% of the extended oligo forms [22].

Among monoJIA, we observed that most patients were in complete remission and among those with AD or CRM, two thirds were on DMARDs for the ocular involvement. These data suggest that the presence of uveitis negatively influences the long-term outcome of monoJIA.

Conversely, among oligoJIA patients, joint involvement remains the major problem in most patients (56%), being arthritis still active, at the last evaluation, in one out of seven.

The fact that monoJIA is a less severe condition was also suggested by the treatment performed during the long-term follow up. In most cases, monoJIA could be managed with IACS that represents the first-line treatment [23–27]. On the contrary, the need to resort to second level drugs (csDMARD or bDMARD) was significantly higher in oligoJIA (over 80% of cases) as an expression of a more severe and more difficult to treat disease [6, 28].

As for patients with initial single joint onset that evolved towards an oligoarticular course, the most relevant aspect that emerged was that this swithches happens within the first three years since the disease onset. Based on this evidence, we can state that, if a patient has arthritis limited exclusively to one joint for at least three years, the probability that he/she will subsequently evolve towards an oligoarticular involvement is extremely low. On the contrary, it is quite probable this patient will maintain a monoarticular course. This also means that, although monoarticular onset is a fairly frequent occurrence in JIA [29], it is necessary to follow the disease for at least three years before classifying it as monoJIA. Therefore, as a result of this *proof-of-concept study*, a possible definition of monoJIA should include the following criteria: arthritis of unknown origin with onset before the age of 16 years with involvement of only one joint, after the exclusion of other conditions [30–33], for a period greater than three years after onset.

Monoarticular JIA has been rarely reported in the literature as isolate condition and mostly as case reports [34, 35] or small case series [36]. In 1965, Bywaters and Ansell, widely considered the founders of pediatric rheumatology in Europe, were the first to describe, within a population of patients with JIA, a homogeneous group of 33 patients with single-joint localization [10]. It is interesting to note how our study, almost 60 years later but with a much larger number of patients and a significantly longer follow-up, reached the same results and conclusions (Table 2). In the latter study fourteen patients had persistent monoJIA course for a follow up period of 3–14 years (mean 6.5), and in 11 (78.6%) the disease was inactive at the last follow-up. Taken together, these data comfort us on the robustness of our observations and confirm that monoJIA may be considered as a distinct clinical entity from oligoJIA.

**Table 2:** Monoarticular JIA: comparison of two series, 60 years apart

		Bywaters Ansell^9^ 1965 No.33	Present series 2023 No. 196	*p*
**Sex** (F, %)		60.6	79.5	*0.289*
**Age at onset** (mean, years)		5.7	5.6	*> 0.999*
**Switch to OligoJIA** (%)		33.3	39.8	*0.498*
**Time to switch** (mean, months)		13.7	17.8	*0.111*
**Uveitis** (%)		14.3	14.4	*> 0.9999*
**Follow up** (mean, years)		6.5	10.4	***0.001***
**Joint involvement** (%)	Lower limbs	93.9	94.1	*> 0.999*
	Knee	69.7	83.9	*0.067*
	Ankle	15.5	10.2	*0.532*
	Wrist	6.0	1.7	*0.208*

Data are number or %

Defining monoJIA as a new and separate subtype of JIA may foresee a possible change in the therapeutic approach which, in addition to the consolidated IACS treatment, may also include alternative treatment modalities in the most severe or refractory cases, such as arthroscopic synovectomy [37, 38] or radiosynoviorthesis, already used in adult RA [39, 40] and in the pediatric age albeit in different conditions such as emophilia-related arthropathy [41], CACP syndrome [42] or others [43, 44].

A limitation of our study lies in its retrospective nature. However, the study included patients followed with a standardized protocol for many years which allowed the clinical trajectory to be followed in detail.

Strengths are the large patients’ sample considered and their careful classification, the multidisciplinary involvement of rheumatologists and ophthalmologists of the same Center and the limited geographical mobility of the subjects which made it possible to analyze their long-term outcome in real time.

## Conclusions

The present proof-of-concept study brings clear evidence that monoJIA presents distinctive features and may be considered as a separate clinical entity from oligoJIA. Our observation may stimulate further studies and also contribute to the large international debate that is attempting to develop a new classification system for JIA, more consistent with the clinical reality [45].

### Electronic supplementary material

Below is the link to the electronic supplementary material.

Supplementary Material 1

## Data Availability

The datasets generated and/or analyzed during the current study are not publicly available (contains patients information).

## Abbreviations

ANA: antinuclear antibodies
F: Female
M: Male
JIA: juvenile idiopathic arthritis
BJH: benign joint hypermobility
RF: Rheumatoid Factor
monoJIA: monoarticular JIA
oligoJIA: oligoarticular JIA
IACS: intraarticular corticosteroid injection
AIDs: anti-inflammatory drugs
csDMARDs: conventional synthetic disease-modifying anti-rheumatic drugs
bDMARDs: biological disease-modifying anti-rheumatic drugs.
