# Supplementary material for: Monoarticular juvenile idiopathic arthritis as a distinct clinical entity A proof-of-concept study
Source: Pediatr Rheumatol Online J. 2023 Aug 10;21:81. doi: 10.1186/s12969-023-00869-w (PMC10416450; doi:10.1186/s12969-023-00869-w)
Supplement: Supplementary file 1 — Supplementary Material 1 [file 12969_2023_869_MOESM1_ESM.doc]

Prof Alberto Martini,
Prof Charles Spencer,

Editors *of Pediatric Rheumatology*

March 18, 2023

Dear Editors,

Please find enclosed the manuscript entitled:

***“******Monoarticular Juvenile Idiopathic Arthritis as a distinct clinical entity***

***A proof-of-concept study”***

that we would like you to consider for publication in *Pediatric* *Rheumatology* as *Research Article*.

Currently, Monoarticular Juvenile Idiopathic Arthritis (monoJIA) is considered as part of the oligoarticular subtype (oligoJIA) in the ILAR Classification although various aspects, coming from the clinical practice, suggest it as a separate entity.

We report the results of a long-term follow up study on the clinical characteristics of a large series of patients with persistent monoJIA. In comparison with oligoJIA, the most significant features of monoJIA are the later onset, reduced female prevalence, higher frequency of joint hypermobility, lower frequency of uveitis and ANA+ and better long-term outcome.

The present proof-of-concept study brings clear evidence that monoJIA presents distinctive features and may be considered as a separate clinical entity from oligoJIA.

Our observation may stimulate further studies and also contribute to the large international debate that is attempting to develop a new classification system for JIA.

All Authors certify that:

- the manuscript is being submitted only to *Pediatric Rheumatology,* will not be submitted elsewhere, while under consideration and has not been published elsewhere
- all authors are responsible for reported research and certify that the material contained herein has not been submitted or published elsewhere
- all authors have participated in the concept and design, analysis and interpretation of data, and drafting or revising of the manuscript, and have approved the manuscript as submitted.

With best regards,

Sincerely,

Francesco Zulian, M.D.
